# Antivenin plants used for treatment of snakebites in Uganda: ethnobotanical reports and pharmacological evidences

**DOI:** 10.1186/s41182-019-0187-0

**Published:** 2020-02-11

**Authors:** Timothy Omara, Sarah Kagoya, Abraham Openy, Tom Omute, Stephen Ssebulime, Kibet Mohamed Kiplagat, Ocident Bongomin

**Affiliations:** 1 Department of Chemistry and Biochemistry, School of Biological and Physical Sciences, Moi University, Uasin Gishu County, Kesses, P.O.Box 3900-30100, Eldoret, Kenya; 2 Department of Quality Control and Quality Assurance, Product Development Directory, AgroWays Uganda Limited, Plot 34-60, Kyabazinga Way, P.O. Box 1924, Jinja, Uganda; 3 Department of Chemistry, Faculty of Science, Kyambogo University, P.O. Box 1, Kampala, Uganda; 4 Department of Quality Control and Quality Assurance, Product Development Directory, Kakira Sugar Limited, P.O. Box 121, Jinja, Uganda; 5 Department of Paediatric and Child Health, Faculty of Medicine, Gulu University, P.O.Box 166, Gulu, Uganda; 6 Department of Biochemistry, Faculty of Health Sciences, Lira University, P.O. Box 1035, Lira, Uganda; 7 Directorate of Government Analytical Laboratory, Ministry of Internal Affairs, P.O. Box 2174, Kampala, Uganda; 8 Department of Mechanical Engineering, School of Engineering, Moi University, Uasin Gishu County, Kesses, P.O. Box 3900-30100, Eldoret, Kenya; 9 Department of Manufacturing, Industrial and Textile Engineering, School of Engineering, Moi University, Uasin Gishu County, Kesses, P.O. Box 3900-30100, Eldoret, Kenya

**Keywords:** Antiophidic, Antivenin, Snakebite, Traditional medicine, Uganda

## Abstract

Snakebite envenomation is a serious public health concern in rural areas of Uganda. Snakebites are poorly documented in Uganda because most occur in rural settings where traditional therapists end up being the first-line defense for treatment. Ethnobotanical surveys in Uganda have reported that some plants are used to antagonize the activity of various snake venoms. This review was sought to identify antivenin plants in Uganda and some pharmacological evidence supporting their use. A literature survey done in multidisciplinary databases revealed that 77 plant species belonging to 65 genera and 42 families are used for the treatment of snakebites in Uganda. The majority of these species belong to family Fabaceae (31%), Euphorbiaceae (14%), Asteraceae (12%), Amaryllidaceae (10%) and Solanaceae (10%). The main growth habit of the species is shrubs (41%), trees (33%) and herbs (18%). Antivenin extracts are usually prepared from roots (54%) and leaves (23%) through decoctions, infusions, powders, and juices, and are administered orally (67%) or applied topically (17%). The most frequently encountered species were *Allium cepa*, *Carica papaya*, *Securidaca longipedunculata*, *Harrisonia abyssinica*, and *Nicotiana tabacum*. Species with global reports of tested antivenom activity included *Allium cepa, Allium sativum, Basella alba*, *Capparis tomentosa, Carica papaya, Cassia occidentalis, Jatropa carcus, Vernonia cinereal, Bidens pilosa, Hoslundia opposita, Maytensus senegalensis, Securinega virosa*, and *Solanum incanum.* There is need to identify and evaluate the antivenom compounds in the claimed plants.

## Introduction

Snake envenoming is a global health problem and a justification for morbimortality and various socio-economic losses. A recent conservative global estimate points that about 5.5 million snakebite cases are encountered every year causing about 2 million deaths [1, 2]. Up to 500,000 of these cases are reported in Africa [3–5]. In 2002, 108 cases of snakebites were reported in Gulu Regional Hospital (Uganda) though none of the victims died [6]. About 151 cases were reported in neighboring Kenya in 1994 with 19% of these from venomous snakes [7].

A recent study [8] in 118 health facilities throughout Uganda revealed that only 4% of the facilities stocked antivenin sera, thus most victims rarely seek medical care when bitten by snakes. A retrospective part of this study showed that in 140 surveyed facilities, 593 snakebite cases were recorded within six months with bites reported in the rainy seasons from April 2018 to June 2018 and then October 2018 to December 2018 [8]. Thus, fatalities in rural areas are due to lack of antidotes within the 24 h recommended [6, 9, 10] and antisera administration problems [11, 12].

Snakes are taxonomically carnivorous vertebrates of class Reptilia, order Squamata, sub-order Serpentes and families**:** Colubridae**,** Boidae**,** Elapidae, Pythonidae**,** Viperidae that characteristically kill their prey by constriction rather than envenomation [13, 14]. Most bites are due to circumstantial stepping on the snakes by unprotected or barefooted victims [6, 15], snake ecology [16] while others are initiated by malevolent and alcohol-intoxicated victims [17–19]. Over 3500 species of snakes have been classified and about 600 (15–17%) of these are venomous [1, 20]. East Africa is home to about 200 species of snakes and 145 of these from 45 genera and 7 families are found in Uganda [21]. Many are harmless or are a rarity though the puff adder (*Bitis arietans*), Gabon viper (*Bitis gabonica*), green or Jameson’s mamba (*Dendroaspis jamesoni***),** black mamba (*Dendroaspis polylepis*), forest cobra (*Naja melanoleuca*), and black-necked spitting cobra (*Naja naja nigricollis*) are listed as venomous [10, 22].

Snake venom is secreted by snake oral glands and is injected subcutaneously or intravenously through the fangs into the victim on the hands, feet, arms, or legs [23]. Venoms are water-soluble, acidic, and have a specific gravity of about 1.03 [24]. The quantity, lethality, and composition of venoms vary with the age and species of the snake, time of the year, geographic location as well as the envenoming snake’s diet. A snake venom is a complex mixture of toxic proteins such as cardiotoxins, neurotoxins, metalloproteinases, nucleotidases, phospholipases A_2_, serine proteinases, acetylcholinesterase nitrate, hyaluronidases_,_ phosphomonoesterase and phosphodiesterase [25] which are injected to immobilize the victim [10, 26]. The toxins cause haemotoxicity-damage to blood vessels resulting in spontaneous systemic and muscle paralysis, myolysis, arrhythmias, cardiac, and renal failure [6].

At present, serum antivenom immunotherapy is the mainstay of treatment reported for snake envenomation [6, 10, 17, 26]. Antisera are either derived from horse serum after injecting it with sublethal doses of the venom (Antivenin Polyvalent) or sheep serum (Crotalidae Polyvalent Immune Fab) [19]. Though antivenom serum is lifesaving, it is associated with the development of immediate or delayed hypersensitivity (anaphylaxis or serum sickness) and does not prevent local tissue damage. The side effects are thought to be due to the action of non-immunoglobulin proteins present in high concentrations in antisera [27]. Worse still, there is a paucity of snake venom antiserum in rural Africa that even in the presence of money, it may not be readily available for purchase [6, 17]. This is in part attributed to the decline in antivenom production in Africa due to denationalization of the manufacturing industries by African countries [28], lack of ready market and low profits from the business. Thus, several attempts have been made to develop snake venom antagonists from other sources including plants, dogs, rabbits, camelids, and avian eggs [12, 27, 29–33].

The use of plants in addressing medical challenges have been witnessed since antiquity and is regaining shape in the modern era due to their safety, effectiveness, cultural preferences, inexpensiveness, abundance, and availability. In Uganda, more than 230 species of angiosperms belonging to about 168 genera and 69 families are being utilized for treatment of erectile dysfunction, malnutrition, sickle cell anemia, hernia, venereal diseases (syphilis, HIV, and gonorrhoea), post-partum hemorrhage, snakebites, cancer, menorrhagia, threatened abortion, skin diseases, jaundice, and cough [34–60]. This study compiled information on antivenin plants reported in different districts of Uganda and presented some experimental evidence supporting their use in antivenom therapy.

## Methodology

### Description of the study area

**Fig. 1:**
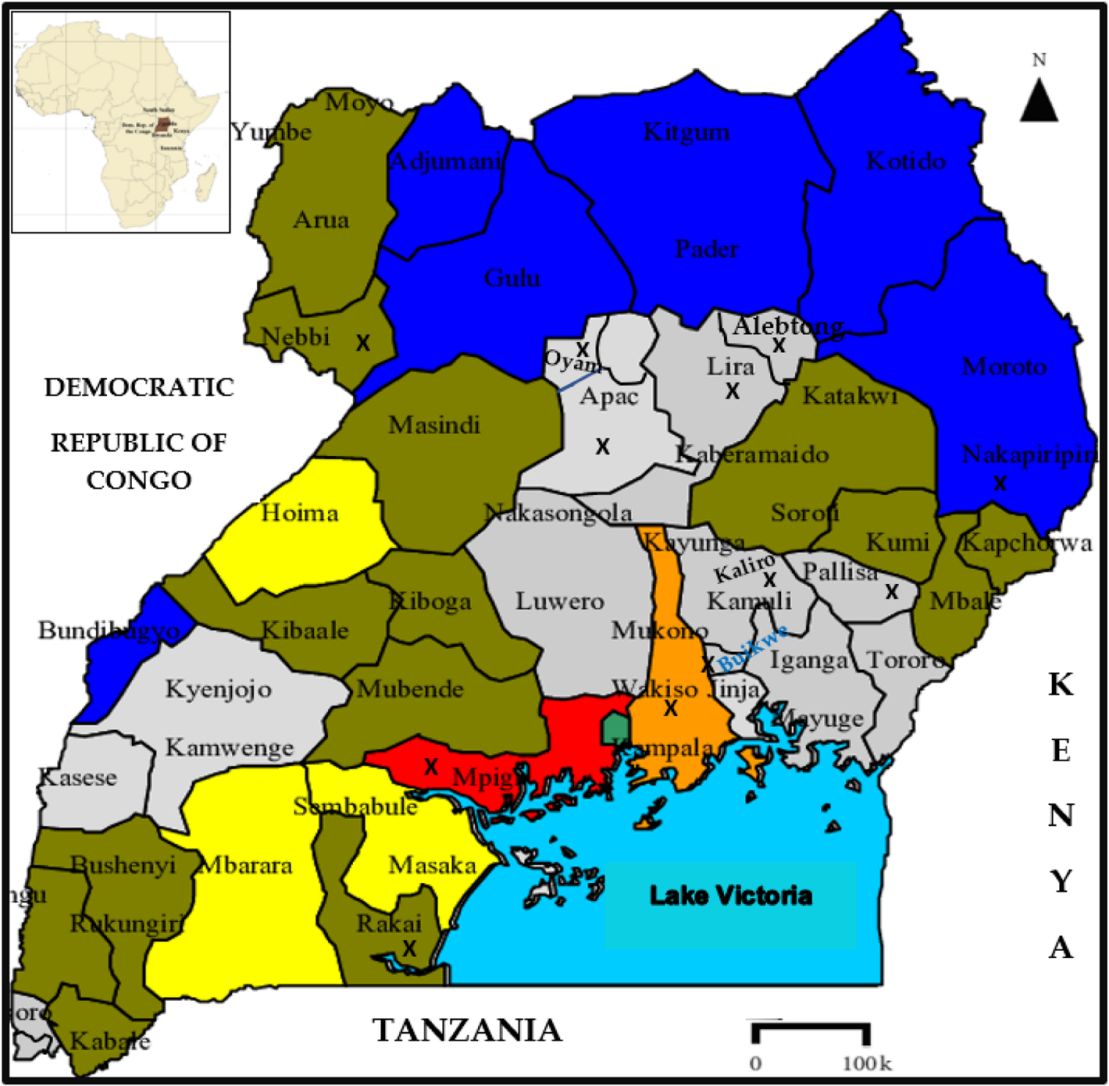
Map of Uganda showing the location of the districts with reports of ethnobotanical surveys (marked X). Inset is the location of Uganda on the African continent

Uganda is a landlocked country straddling the equator in Eastern Africa [61]. It is flanked by Lake Victoria, Tanzania, and Rwanda to the south, Kenya to the East, South Sudan to the North and Democratic Republic of Congo to the West (Fig. 1). The climate experienced is equatorial moderated by relatively high altitudes with a mean annual temperature of 20.5 °*C*. The country’s population is estimated to be 35.92 million with 5 main ethnic families: Nilotics (Acholi, Alur, Padhola, Lulya, and Jonam), Bantu (Baganda, Banyankole, Batoro, Bagwere, Bakiga, Bakiga, Banyarwanda, Bakonjo, Banyoro, and Bakiga), Hamities (mainly constituted by the Bahima), the Nilo-Hamities (Teso, Karamojong, Kakwa, Sebei, Labwor, and Tepeth) and the Sudanics (Lugwara, Madi, and Lendu) [62]. Health care services are inadequate [63], and access to allopathic drugs is limited in rural areas due to their prohibitive cost, poor transport network, chronic poverty and the general belief in efficacy of traditional medicine than western medicine [64].

### Literature search strategy

Relevant original articles, books, thesis, dissertations, patents, and other reports written in English and other local languages on ethnobotany and pharmacological evidences on snakebites in Uganda were searched in Scopus [65], Web of Science [66], PubMed [67], Science Direct [68], Google Scholar [69], and Scientific Electronic Library Online (SciELO) [70] from July 2019 to September 2019. The key search words used were “snakebite,” “vegetal,” “traditional medicine,” “ethnobotany,” “alternative medicine,” “ethnopharmacology,” “antivenom,” “antiophidian,” “antitoxin,” “snake antidotes,” and “Uganda.” The botanical names of the plants were vetted in botanical databases: the Plant List [71], International Plant Names Index (IPNI) [72], NCBI taxonomy browser [73], and Tropicos [74]. Where a given species was considered as distinct species in different reports, the nomenclature as per the botanical databases took precedence. The families, local names (Lango, Acholi, Ateso, Luganda, Lunyoro, Rukiga, and Lusoga), growth habit, part(s) used, conservation status, preparation and administration mode, status of antivenin activity investigation of the plants, and the districts where the plants were surveyed are reported (Table 1, Additional file 1). Pertaining to pharmacological reports, the snake venom studied, phytochemicals, and positive results obtained using plants identified by this study (or species from the same genus) are reported. In some cases, some activities of the plant extracts such as antioxidant and radical scavenging activities are reported as these are some of mechanisms by which snake venoms are countered.

**Table 1:** Antivenin plants used in rural communities of Uganda

Plant family	Latin botanical name	References
Acanthaceae	*Asystasia schimperi* T. Anders.	[42]
Amaryllidaceae	*Allium cepa* L.	[41, 42, 49]
Amaryllidaceae	*Allium sativum* L.	[49]
Amaryllidaceae	*Crinum kirkii*	[41]
Amaryllidaceae	*Scadoxus multiflorus* (Martyn) Raf.	[10, 42]
Apocynaceae	*Carrisa edulis*	[50]
Apocynaceae	*Thevetia peruviana* (Pers.) Schumann	[42]
Aristolochiaceae	*Aristolochia tomentosa* Sims.	[50]
Aristolochiaceae	*Aristolochia elegans* Mast.	[42]
Asclepiadaceae	*Cryptolepis sanguinolenta* (Lindl.) Schltr	[42]
Asparagaceae	*Sansevieria dawei* Stapf	[38]
Asparagaceae	*Sansevieria trifasciata* var. trifasciata	[10]
Asteraceae	*Bidens pilosa* L.	[42]
Asteraceae	*Crassocephalum mannii* (Hook.f.) Milne-Redh.	[35]
Asteraceae	*Echinops amplexicaulis* Oliv.	[46]
Asteraceae	*Microglossa pyrifolia* (Lam.) O. Kuntze	[42]
Asteraceae	*Vernonia cinerea* (L) Less	[41, 42]
Basellaceae	*Basella alba* L.	[39]
Boraginacea	*Trichodesma zeylanicum* (L.) R.Br.	[41]
Cleomaceae	*Cleome gynandra* L.	[35]
Capparidaceae	*Capparis tomentosa* Lam.	[42]
Caricaceae	*Carica papaya* L.	[41, 42, 50]
Celastraceae	*Maytensus senegalensis* (Lam) Exell.	[41]
Combretaceae	*Combretum collinum* Fresen	[41]
Combretaceae	*Combretum molle* ex G.don.	[41]
Commelinaceae	*Murdannia simplex* Vahl. Branan	[35]
Compositae	*Aspilia africana* C.D Adams	[46]
Convolvulaceae	*Hewittia sublobata* L. Kuntze	[49]
Convolvulaceae	*Ipomoea batatas* (L.) Lam.	[42]
Dracaenaceae	*Dracaena steudneri* Engl.	[49]
Ebenaceae	*Euclea divinorum* Hiern	[42]
Euphorbiaceae	*Acalypha bipartita* Muell. Arg.	[42, 47]
Euphorbiaceae	*Croton macrostachyus* Hochst. ex. Delile	[49]
Euphorbiaceae	*Euphorbia tirucalli* L.	[35]
Euphorbiaceae	*Jatropha curcas* L.	[42]
Euphorbiaceae	*Ricinus communis* L.	[35, 42]
Euphorbiaceae	*Securinega virosa* (Willd) Baill.	[41]
Fabaceae	*Acacia seyal* Del. var. *fistula* (Schweinf.) Oliv.	[42]
Fabaceae	*Acacia* species	[42]
Fabaceae	*Albizia coriaria* (Welw. ex) Oliver	[42]
Fabaceae	*Canavalia ensiformis* L. D.C	[10]
Fabaceae	*Indigofera arrecta* Host. A. Rich.	[42, 49]
Fabaceae	*Indigofera garckeana* Vatk	[42]
Fabaceae	*Indigofera capitata* Forsk.	[41]
Fabaceae	*Pseudarthria hookeri* Wight and Arn.	[42, 48]
Fabaceae	*Senna occidentalis* (L.) Link	[42]
Fabaceae	*Senna septemtrionalis* (Viv.) I. et B.	[39]
Fabaceae	*Senna siamea* (Lam.) Irwin and Barneby	[42]
Fabaceae	*Senna singueana* (Del.) Lock	[42]
Lamiaceae	*Hoslundia opposita* Vahl	[42]
Lamiaceae	*Plectranthus barbatus*	[37, 50]
Leguminosae	*Cassia occidentalis* L.	[35]
Liliaceae	*Anthericum cameroneii* Bak	[41]
Loganiaceae	*Strychnos innocua* Del.	[41]
Malvaceae	*Urena lobata* L.	[42]
Melastomataceae	*Tristemma mauritianum* J.F. Gmel.	[41]
Meliaceae	*Ekebergia capensis* Sparrm	[44]
Meliaceae	*Trichilia ematica* Vahl	[38, 46]
Menispermaceae	*Cissampelos muchronata* A.Rich.	[41, 49]
Moraceae	*Ficus natalensis* Hochst.	[42]
Myricaceae	*Morella kandtiana* (Engl.) Verdic and Polhill	[49]
Papillionaceae	*Ormocarpum trachycarpum*	[50]
Passifloraceae	*Adenia cissampeloides* (Hook.) Harms	[42]
Poaceae	*Imperata cylindrica* (L.) P. Beauv	[42, 49]
Poaceae	*Sporobolus pyramidalis* P. Beauv.	[42]
Polygalaceae	*Securidaca longipedunculata* Fres.	[41, 42, 50]
Rosaceae	*Rubus rigidus* Sm	[49]
Rubiaceae	*Gardenia ternifolia* Schumach. and Thonn.	[42]
Rutaceae	*Citrus sinensis* (L.) Osb.	[42]
Rutaceae	*Fagaropsis angolensis* (Engl.) Dale	[59]
Simaroubaceae	*Harrisonia abyssinica* Oliv.	[41, 42, 50]
Solanaceae	*Datura stramonium* L.	[41]
Solanaceae	*Nicotiana tabacum* L.	[42, 49, 59]
Solanaceae	*Solanum aculeatissimum* Jacq	[41, 46]
Solanaceae	*Solanum incanum* L.	[41, 42]
Umbifellifereae	*Steganotaenia araelicea* Hoscht	[41]
Verbenaceae	*Lantana camara* L.	[50]

### Results and discussion

Only full-text articles in English, Lango, Acholi, Ateso, Luganda, Lunyoro, Rukiga, and Lusoga were considered. A total of 15 articles (13 in English, 1 in Luganda, and 1 in Lusoga) with information on antivenin plants were retrieved, but two of these did not meet inclusion criteria because one was not a full-text article while the other had only one botanically unidentified antivenin plant. Thus, the following reports of interest specifically on the subject of antivenin plants in Uganda were retrieved (Table 1).

### Traditional concept of snakebites in Uganda

From the electronic survey of data, it is indubitable that the local communities in Uganda have different perceptions about snakebites. The beliefs appear to be clan-related and include snakes “can protect” (among the Baganda) [18, 75] or “are dangerous and connected to witchcraft” in most communities [8]. By comparison, the Luo of Kenya associate snakes with witchcraft [76].

**Fig. 2:**
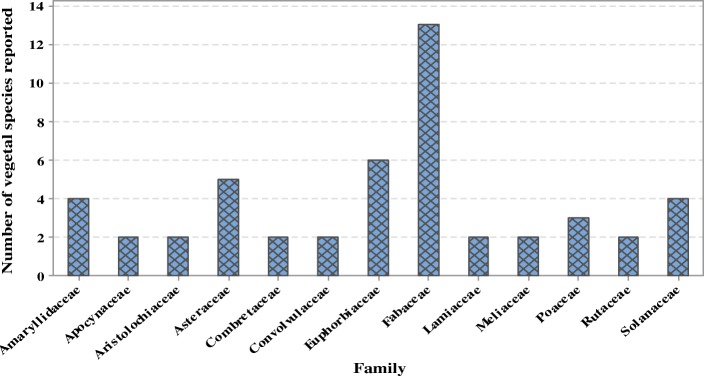
Major families from which vegetal antivenins are obtained in Uganda

From the survey, 77 plant species from 65 genera belonging to 42 botanical families claimed as antiophidic in Uganda were retrieved (Table 1, Additional file 1). The most cited families were Fabaceae (31%), Euphorbiaceae (14%), Asteraceae (12%), Amaryllidaceae (10%), and Solanaceae (10%) (Fig. 2). Most families encountered in this study have reported antivenin potential in treating or avoiding snakebites in other countries across the globe. For example, Apocynaceae, Aristolochiaceae, Asteraceae, Convolvulaceae, Fabaceae, and Myricaceae were cited in Kenya [17] and Tanzania [77], Meliaceae in Ghana [78], Fabaceae in Rwanda [79], Asparagaceae, Leguminosae, and Menispermaceae in Sudan [80], Acanthaceae, Apocynaceae, Asteraceae, Capparaceae, Cariaceae, Combretaceae, Convulaceae, Ebenaceae, Eurphorbiaceae, Fabaceae, Malvaceae, Meliaceae, and Poaceae in Ethiopia [81] and Pakistan [82], Fabaceae, Aristolochiaceae, and Lamiaceae in Djibouti [83] and Nigeria [84], Melastomataceae and Menispermaceae in Cameroon [85]. Acanthaceae, Apocynaceae, Asteraceae, Euphorbiaceae, Fabaceae, Moraceae, Rubiaceae, and Rutaceae were cited in India [86, 87], Bangladesh [88, 89], and Central America [90]. Fabaceae is always dominant in ethnobotanical reports because of the abundance of plant species from this family [88, 91–93].

**Fig. 3:**
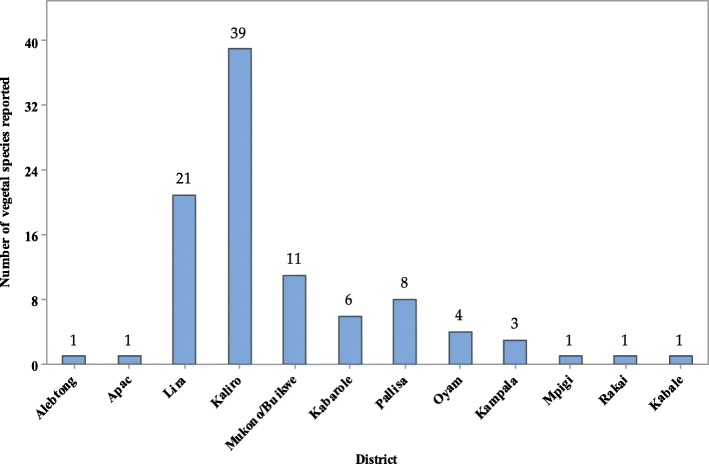
Distribution of antivenin plant species in Ugandan districts as per ethnobotanical reports

The families reported were from different districts of Uganda (Fig. 3) representing different ethnic groups with diverse cultural beliefs and practices. About 40% of the plant species were reported in Kaliro (inhabited by the Basoga) followed by 21% from Lira (occupied by the Lango) and 11% from Mukono-Buikwe frontier occupied by the Baganda. In a similar cross-cultural comparison of antiophidic floras in the Republic of Kenya, Owuor and Kisangu [17] reported that two culturally and floristically distinct African groups (Kamba and Luo) had similar knowledge of snakebites but the antivenin plants utilized by the two ethnic groups were independently derived. The abundance of antivenin plants from Kaliro, Lira, and Mukono/Buikwe could be due to the presence of forest reserves in these districts. Kaliro, Namalemba, and Namukooge local forest reserves are found in Kaliro [94]. The district is also rich in water resources such as Lake Nakuwa, River Mpologoma, Naigombwa, and Lumbuye wetlands which provide rainfall for the growth of plants. Lira District has Lake Kwania, Okole, Moroto and Olweny wetland systems which support the growth of plants [95]. The district gazetted over 1000 hectares of land for forest conservation and this serves as a good source of plants for traditional medicine [96]. The Mukono-Buikwe frontier has Mabira forest reserve which has been protected since 1932 and contains a number of endangered plant species in Uganda [97]. The rainforest is a rain catchment for areas supplying River Nile and Ssezibwa River and has rainfall throughout the year thus plants flourish in this area [98].

### Growth habit, parts used, preparation, and administration of antivenin preparations

**Fig. 4:**
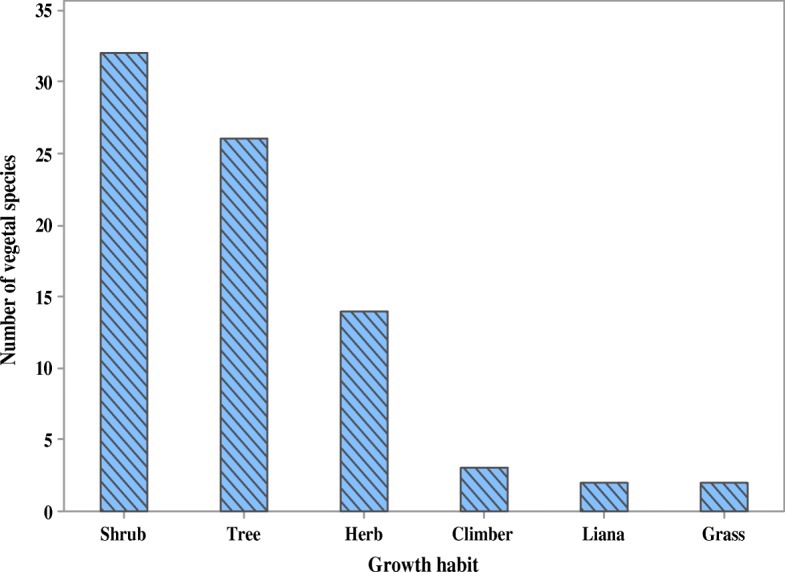
Growth habit of the antivenin plants used in rural communities of Uganda

**Fig. 5:**
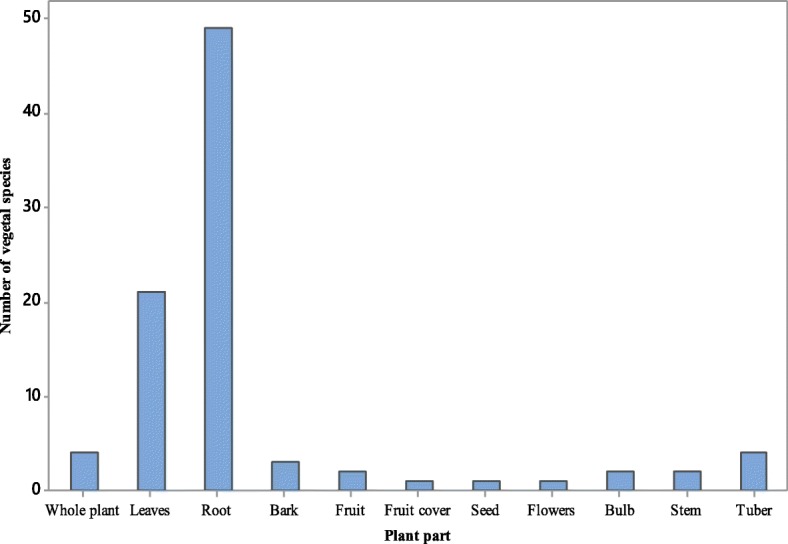
Parts of antivenin plants used in rural communities of Uganda

Antivenin plants used in Uganda are majorly shrubs (41%), trees (33%) and herbs (18%) and the commonly used plant parts are roots (54%) and leaves (23%) followed by whole plant (4%), bark (4%), and tuber (4%) (Figs. 4 and 5). The regular use of roots and leaves in antivenin preparations is a characteristic feature of traditional antivenin therapy [17, 81, 86, 99, 100], no wonder some of these plants are named “snakeroot” in some rural communities [101]. Comparatively, embryonal plant parts such as fruits, seeds, buds, bulbs, and flowers which have reputation for accumulating certain compounds are less frequently used, concordant with reports from other countries [17, 81]. Majority of the plants reported grow in the wild (82%), 14% are cultivated while 4% are semi-wild (occurs in the wild but can also be cultivated). The commonest mode of preparation is as decoctions and infusion. The plants are collected from fallow land, cultivated fields or home gardens when needed. Traditional medicine practitioners either collect herbal plants personally or hire collectors. All traditional medical practitioners cultivate some medicinal plants especially fast growing ones around their homes and shrines in order to have them within easy access when needed. The antidotes are administered orally (67%) or applied at the point of snakebite (17%).

In this survey, it was noted that few plant species are used against snakebites simultaneously in different districts. This could probably be attributed to the abundant distribution of the analog active substances among species especially those of family Fabaceae. Some of the plants listed are also used for wading off or discouraging snakes from reaching human and livestock abodes. In most instances, the plants possess a strong smell that causes discomfort and disorientation to snakes when they slither over them. In exceptional cases as with tobacco, the plant (dried whole plant or leaves) are burnt to produce unpleasant odor that discourages snakes (Table 2). The Lango of Northern Uganda burn bicycle, motorcycle, and vehicle tyres to discourage snakes.

**Table 2:** Plants used in Ugandan rural communities for repelling of snakes

Family	Botanical name	Growth habit	Part used	Mode of use to prevent snakes	References
Amaryllidaceae	*Allium cepa* L	Herb	Bulb	Decoction made and sprinkled around the house. Snakes are discouraged by the sharp onion smell.	[10]
Amaryllidaceae	*Allium sativum* L.	Herb	Bulb	Decoction made and sprinkled around the house. Snakes do not are discouraged by the sharp onion smell.	[10]
Asteraceae	*Tagetes minuta*	Herb	Leaves	Plants have bitter tastes and strong smells that cause discomfort and disorientation to snakes when they slither over them.	[10]
Euphorbiaceae	*Ricinus communis*	Herb	Leaves/whole plant	Plant have strong smell that cause discomfort and disorientation to snakes when they slither over them.	[10]
Poaceae	*Cymbopogon citrus*	Grass	Leaves	Decoction made and sprinkled around the house. Snakes do not like the citrus smell from the leaves	[10]
Solanaceae	*Nicotiana tabacum* L.	Shrub	Leaves	Planted around the house, leaves burnt	[10, 102]

### Other ethnomedicinal uses and toxicity of the reported antivenin plants

Almost all the plants recapitulated in this review are employed for the treatment of various ailments. For example, *Bidens pilosa* L. has been reported to be useful in the treatment of more than 40 disorders including inflammation, immunological disorders, digestive disorders, infectious diseases, cancer, metabolic syndrome, and wounds among others [103–106]. *Albizia coriaria* (Welw. ex) Oliver is used in the management of syphilis, post-partum haemorrhage, sore throats, menorrhagia, threatened abortion, skin diseases, jaundice, cough, sore eyes, and as a general tonic [35]. Such plants tend to be used in different communities for treating snakebites and can be a justification of their pharmacological efficacy [107].

On the other hand, some of the antivenin plants cited exhibit marked toxicity. A striking example is *Jatropha carcus* L*.* leaf and latex which contain a purgative oil (irritant curcanoleic acid and croton oil), curcin (toxalbumin), and diterpene of tigliane skeleton classified as phorbol esters [108]. Curcin has protein translation inhibitory (*N*-glycosidase) activity whereas phorbol esters are amphiphillic molecules that can bind phospholipid membrane receptors [109]. This observation explains why some antivenin preparations in Uganda are applied topically or ingested in small amounts. Fortuitously, topical application is a better approach for reducing the local action of venoms at the bitten site.

### Knowledge dynamics of antivenin plants in Uganda

Knowledge of traditional medicine and medicinal plants are usually acquired and passed on orally from the elders to the young [34]. This is comparable to reports from other African countries [17, 78]. Knowledge is gained through trainings, divine call, and in some instances, the plant to be used can be asked for from the dead [42, 59]. Because of civilization, efforts to pass on traditional medical knowledge to children is impeded by lack of interest and the fact that most children spend their youthful years in school [17, 34, 60]. Most Ugandans know that their current social conditions such as poverty, sleeping in mud houses and activities such as cultivation, hunting, and herding cattle increase their chances of getting bitten by a snake. Snakebites are always taken as exigencies with economic implications due to the expenses involved in transporting the victims for treatment, the care needed, enforced borrowing, amputation of necrosed legs, and arms as well as loss of time [8].

### Treatment of snakebites

Treatment of snakebites in Uganda involves various procedures that vary from culture to culture and religion to religion, for example, Pentecostal Assemblies of God (PAG) believe prayers can treat snakebites. Use of tourniquets to tie the injured part above the affected area to prevent the venom from spreading to heart, the lungs, kidney, and other delicate parts of the body has been prescribed as a supportive first aid in Northern Uganda [6]. This is usually done at five-minute intervals to avoid the weakening of the local tissues.

**Fig. 6.**
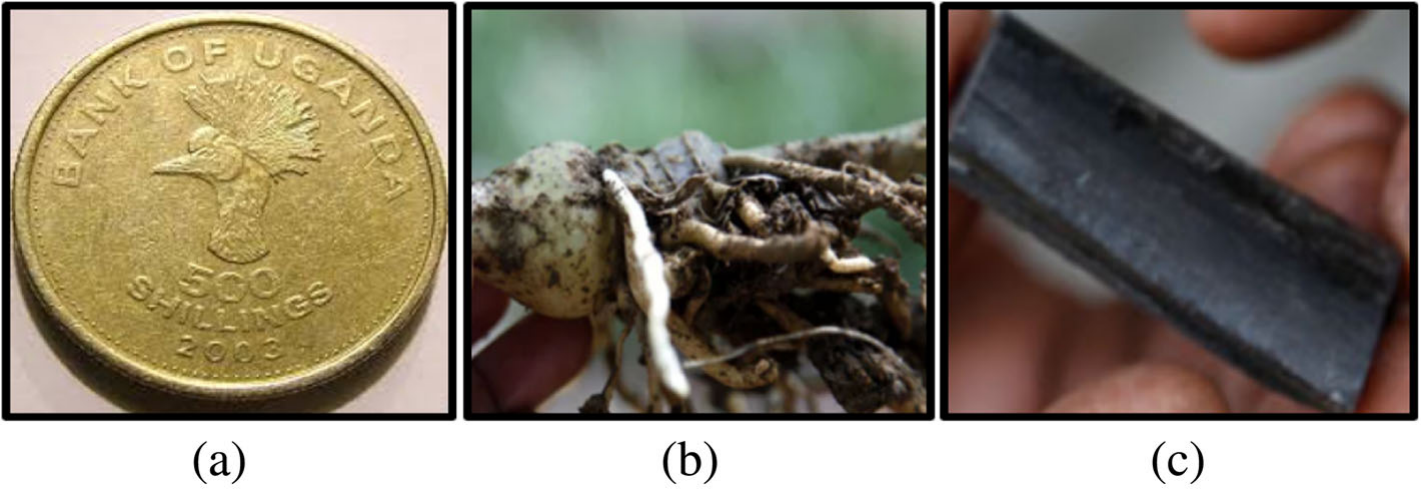
Treatment of snake bites in Uganda. **a** 500 Uganda shillings copper coin. Side displayed is usually placed on the bite. **b***Haemanthus multiflorus* bulb. **c** black stone

Among the Baganda (Central Uganda), the use of black stones (carbonized absorptive animal bone) and *Haemanthus multiflorus* bulb have been reported (Fig. 6) [10]. A black stone is placed on incisions made around the bitten area until it sticks. It is administered to reassured victims and left for 20-30 minutes for it to “suck out” the poison. The stone is reported to be 30% effective and can be reused if boiled in hot water after use and can be used alongside other medical treatments [10]. For *Haemanthus multiflorus*, the bulb is chewed by the victim or it is crushed and put on the bite.

In Northern Uganda, the use of 500 Uganda shilling copper coins and black stones have been reported [6]. The copper coins are placed on the bite until it gets stuck and it is left to fall off on its own. In some communities like Lango of Northern Uganda, antivenin therapy involves oral administration of egg yolk and albumin similar to the therapy reported among the Luo of Kenya [17]. Overall, traditional antivenin therapy in Uganda involves administration of plant preparations to the victims [35].

### Antivenin activity of plants and pharmacological evidence

Pharmacological studies have revealed that some plants used in traditional medicine are able to antagonize the activity of various crude venoms and purified toxins [110–112]. Antigen-antibody interaction is the proposed mechanism through which the activity of venoms is countered by antivenins. Reported mechanisms of venom inactivation include precipitation or inactivation of the toxic venom proteins [113], inactivation, or enzyme inhibition [114], chelation [115], adjuvant action [116], antioxidant activity or a synergistic interaction of these mechanisms. Enzyme inhibition and protein precipitation are by far the most conventionally accepted mechanisms [117]. To start with, plant metabolites such as flavonoids, polyphenols, saponins, tannins, terpenoids, xanthenes, quinonoids, steroids, and alkaloids have been reported to snuggly bind to toxic proteins of snake venoms, thereby offsetting their deleterious effects. Another explained scientific possibility is the competitive blocking of the target receptors [118]. For example, atropine (an alkaloid reported in family Solanaceae) is reported to inhibit the activity of green and dark mamba (*Drendroaspis angusticeps* and *D. polylepsis*) venoms by blocking cholinergic nerve terminals usually attacked by the venoms. Aristolochic acid I (8-methoxy-6-nitro-phenanthro(3,4-d)1,3-dioxole 5-carboxylic acid), an alkaloid present in *Aristolochia* species acts in the same way.

Another mechanism of snake venom inactivation involves inhibition of the active enzymes such as phospholipase A_2_, metalloproteases, and hyaluronidases by polyphenolic compounds such as tannins. In this scenario, the metabolites interact with the venom enzymes by non-specific binding proteins [119] through hydrogen bonding with hydroxyl groups in the protein molecules generating chemically stable complexes [120]. For example, in a study experimented with aristolochic acid I and PLA_2_ isolated from *Viper russelli* venom, molecular interactions between the two were reported to be between their hydroxyl groups which formed two hydrogen bonds with Granulocyte Marker Monoclonal Antibody (His48) and myotoxins I (Asp49) of the venom [121]. Aristolochic acid I is also an inhibitor of hyaluronidase of *Naja naja* venom [122]. Other examples of these are outlined in Table 3. Chelation on the other hand is reported to be effective for antivenin plant extracts with molecules (compounds) capable of binding to divalent metal ions necessary for some enzymatic activities. For the cause that chemical coordination of metal ions is indispensable for normal hydrolytic activities of phospholipases and metalloproteases, secondary metabolites capable of disrupting the enzyme-metal ion bondage inhibits enzymatic progression [166]. In antioxidation mechanism, plant metabolites (flavonoids, terpenoids, tannins, polyphenols, vitamins A, C, E, and minerals such as selenium) prevent, stop or reduce oxidative damage due to phospholipase A_2_ activity by selectively binding to the active sites or modifying the conserved residues that are inevitable for phospholipase A_2_ catalytic action [119].

**Table 3:** Antivenin activities of some plants used for snakebite treatment in Uganda as per global reports

Plant	Part used	Solvent used	Antivenin activity (comments)	Active chemical constituents	Authors
*Allium cepa* L.	Bulb	Methanol	Cardioprotective activity (14.8 ± 1.65 units/l; *p* > 0.5) on creatine kinase isoenzyme levels to neutralize snake venoms. Concentrations (< 160 μg/ml) stabilized human red blood corpuscles membrane (antihemolytic) against *N. naja karachiensis* venom, though elevated concentrations were cytotoxic. Provided 50% protection from *N. naja karachiensis* phospholipase A (PLA_2_) in terms of an increase in pH of an egg yolk suspension. Neutralized the anticoagulant effect induced by weak PLA_2_ enzymes in *N. naja karachiensis* venom (76% inhibition, coagulation time of 106 ± 0.57 s). Quercetin is a potent inhibitor of lipoxygenase	Quercitin, sulfurous volatile oils, oleanolic acid, protocatechuric acid	[123–127]
*Allium sativum* L.	Bulb	Methanol	Hepatoprotective activity (*p* > 0.5, 49 ± 5.01 and 82.5 ± 18.55 units/l of aspartate aminotransferase and alanine aminotransferase against 52.5 ± 3.51 and 69.5 ± 18.55 units/l for standard antiserum) assessed in rabbits. Provided 50% protection from *N. naja karachiensis* PLA_2_ in terms of an increase in pH of an egg yolk suspension. Provided 50% protection from *N. naja karachiensis* PLA_2_ in terms of an increase in pH of an egg yolk suspension. Neutralized the anticoagulant effect induced by weak phospholipase A enzymes in *N. naja karachiensis* venom (40% inhibition, coagulation time of 115 ± 1.52 s).	Quercetin, scordinines A, B allicin, thiosulfinates, 2 mercapto-L-cysteines, anthocyanins, alliinase, polysaccharides, sativin I, sativin II, glycosides of kaempferol	[123, 125, 126]
*Asystasia* spp *(A. gangetica* L)	Leaves	Methanol	1000 mg/kg provided 80% protection against *N. melanoleuca* venom (PLA_2_)	Flavonoids, saponins and tannins	[128]
*Aristolochia* spp (*A. indica, A. odoratissima*)	Leaves	Methanol, Ethanol, Water, pentane	PLA_2_ and hyaluronidase enzymes from *N. naja* and *V. russelli* venoms inhibited. Strong gelatinolytic, collagenase, peroxidase, and nuclease activities, l-amino acid oxidase and protease inhibitory potencies. Protected mice against lethal effects of *Bothrops atrox* venom at higher doses of 8 and 16 mg/kg	Aristolochic acid I, lignan (-)-cubebin	[129–131]
*Basella alba* L.	Fruit	Methanol	Radical scavenging activity against 1,1-diphenyl 2-picrylhydroxyl (DHPP) experimented in mice.	Flavonoids, phenolics, betacyanins, Lupeol, β sitosterol	[132–134]
*Capparis tomentosa* Lam.	Root	Water, petroleum ether	The antioxidant activity by DPPH was 35.50 ± 0.02%, by phosphomolybdate assay was 41.22 ± 0.17 mg/kg ascorbic acid equivalent, and the reducing power increased with increase in concentration up to a maximum at 800 μg/ml in alloxanized male mice (aqueous extracts).	N-benzoylphenylalanylaninol acetate, 24-ethylcholestan-5-en-3-ol, L-stachydrine, 3-hydroxy-3-methyl-4-methoxyoxindole	[135, 136]
*Carica papaya* L.	Leaves	Water, ethanol	Hepatoprotective against carbon tetrachloride induced hepatotoxicity in mice.	Saponins, cardiac glycosides, alkaloids, phenolic acids, chlorogenic acid, flavonoids and coumarin compounds	[137–140]
*Carissa* spp (*C. spinarum* L.)	Leaves	Methanol	Acetylcholinesterase, PLA_2_, hyaluronidase, phosphomonoesterase, phosphodiesterase,5-nucleotidase enzymes from *Bungarus caeruleus* and *V. russelli* venoms inhibited by 100 μg/ml of the extract.	Steroids, flavonoids, tannins, saponins, alkaloids, ursolic acid	[141, 142]
*Cassia occidentalis* L.	Leaves, roots	Ethanol	Stimulated angiogenesis, inhibited epidermal hyperplasia, and minimized local effects caused by *Bootrops moojeni* venom.	Anthraquinones	[143, 144]
*Citrus* spp. (*C. limon* L. Burm. F)	Root, ripe fruits	Methanol	Neutralized the anticoagulant effect induced by weak PLA_2_ enzymes in *N. naja karachiensis* venom (64% inhibition, coagulation time of 109 ± 1.00 s). In vitro inhibitory ability against the lethal effect of *Lachesis muta* venom with effective dose 50% of 710 μg extract per mouse	d-x-pinene camphene, d-limonene, linalool, ichangin 4-β-glucopyranoside, nomilinic acid, 4-β-glucopyranoside	[126, 145, 146]
*Cleome* spp (*C. viscosa*)	Bulb	Methanol, ethyl acetate	Significant anti-inflammatory activity against carageenin-, histamine-, dextran-induced rat paw edema compared to Diclofenac sodium (20 mg/kg) standard	Flavonoid glycosides, querection 3-0-(2″-acetyl)-glucoside, phenolics	[147, 148]
*Crinum* spp (*C. jagus*)	Bulb	Methanol	Extract of 1000 mg/kg protected 50% of mice; injection of a pre-incubated mixture of the same extract dose and venom gave 100% protection against *E. ocellatus* venom (10 mg/kg). Administration of extract at 250 mg/kg, 30 min before the injection of *E. ocellatus* venom (10 mg/kg) prolonged (*p* < 0.05) death time of poisoned mice. Extract of 500 mg/kg provided 50% protection against *Betans* venom (9.5 mg/kg) while pre-incubation of a mixture of the same dose of venom and extract prior to injection provided 33.3% protection. Plasma creatine kinase concentrations in poisoned mice reduced with injection 1000 mg/kg of extract pre-incubated with 5 mg/kg of *E. ocellatus* or 7 mg/kg *B. arietans* venoms. The extract blocked hemorrhagic activity of a standard hemorrhagic dose (2.8 mg/ml) of *E. ocellatus* venom at 1.7, 3.3, and 6.7 mg/ml.	Phenolic compounds, tannins, alkaloids, cardiac glycosides	[148, 149]
*Indigofera* spp. (*I. capitata* Kotschy, *I.* c*onferta* Gillett)	Leaves	Methanol, ethanol, water	Extracts reduced bleeding and clotting times of *N. nigricollis* envenomed rats. Ethanol and aqueous extracts of *I. capitata* were more effective at dose of 300 mg/kg with lowest clotting time of 174 ± 3.67 s and 1000 mg/kg with lowest bleeding time of 228 ± 3.00 s. *I. conferta* at a dose of 1000 mg/kg had the lowest clotting time of 173 ± 5.61 s (ethanol extract) and 234 ± 7.64 s for aqueous extract). Edema forming activity was inhibited by ethanol and aqueous extracts, effective at higher doses of 300 mg/kg (ethanol extract) and 1000 mg/kg (aqueous extract) with the lowest edema forming activity of 108.80 ± 1.90 and 102.00 ± 1.90 (%mm) respectively by *I. capitata* and at dose of 250 mg/kg, 500 mg/kg, and 1000 mg/kg of aqueous extract with the lowest edema forming activities of 100.8 ± 1.89, 100.20 ± 1.90 and 100.60 ± 1.90 (%mm) by *I. conferta*	Flavonoids, phenolic compounds, steroids, triterpenes, anthraquinone, alkaloids	[150]
*(I. pulchra* Willd.)	Methanol		Extract inhibited anticoagulant, hemolytic and PLA_2_ activities of *N. nigricollis* venom	Tannins, flavonoids, saponins, and steroids	[148, 151]
*Jatropa carcus* L.	Leaf latex	Methanol	Inhibits hemolytic activity of PLA_2_ from *N. naja* venom	Terpenoids, alkaloids, phenolics, flavonoids, saponins	[152]
*Vernonia cinerea* (L) Less.	Whole plant	Methanol	Antioxidant activity by DPPH free radical scavenging assay. Ethyl acetate fraction exhibited 63.3% DPPH radical scavenging activity at 100 μg/ml.	Phenolics, flavonoids	[153]
*Sansevieria* spp (*S.* liberica ger. and labr)	Rhizome, root	Methanol	LD_50_ of 353.5 ug/kg. The extract, n-hexane, ethyl acetate, and butanol fractions significantly protected mice from *N. naja nigricollis* venom-induced mortality	Terpenoids, flavonoids, saponins	[154]
*Albizia* spp *(A. lebbeck* L. (Benth) bark)	Root/bark	Water	1000 mg/kg*, N. kauothia* venom, provided 50% protection from *N. naja karachiensis* PLA_2_ in terms of an increase in pH of an egg yolk suspension	Carbohydrates, proteins, alkaloids, flavonoids, tannins, echinocystic acid, amino acids	[109, 123, 125, 154]
*Euphorbia species (E. hirta)*	Whole plant	Methanol	LD_50_ not specified, against *N. naja*) venom	Quercetin-3-O-alpha-rhamnoside, terpenoids, alkaloids, steroids, tannins, flavonoids, phenolic compounds	[155, 156]
*Bidens pilosa* L.	Leaves, whole part	water, hexane	Effective against *Dendroaspis jamesoni* and *Echis ocellatus* venom	Linalool, Cadinene, 훽-Caryophyllene, 훽-Cubebene, Cedrene, Humulene, Selina-3,7(11)-diene, Thujopsene, *(*−*)*-Globulol, Elixene, 2-Hexen-1-ol, 2-Hexenal	[157, 158]
*Hoslundia opposita* Vahl	Root, leaves	Methanol, Water	DPPH radical scavenging activity of 32.3 ± 1.9 μg/ml compared to standard l-ascorbic acid with the activity of 21.1 ± 1.1 μg/ml.	훼-Cadinol Ethyl linolenate, Palmitic acid	[158, 159]
*Maytensus senegalensis*	Root	Methanol, chloroform	Anti-inflammatory activity inhibited ear edema induced by croton oil in mice	Maytenoic acid, lupenone, *β*−amyrin	[160]
*Securinega virosa*	Leaves	Hexane, ethyl acetate, methanol	N-hexane extract provided protection against lethal dose of *Naja nigricollis* venom (significant at 20 mg/kg, *p* < 0.05)	Alkaloids, phenols, saponins and triterpenes/steroids	[161, 162]
*Solanum incanum* L.	Root	Water	Inhibited the response to acetylcholine in a concentration-dependent manner like atropine. The extract inhibited charcoal travel in mice intestine by 36.28, 51.45, 52.93, and 38.53% in doses of 50, 100, 200, and 400 mg/kg body weight respectively	Quercetin, Isoquercitrin, Kaempferol, β-Sitosterol, Luteolin 7-O-b-D-glucopyranoside, sodium, potassium, chromium, vitamins B and C	[162–165]

The efficacy of plant extracts in antivenom action tends to be related to the solvent used for the extraction of the bioactive compounds. A study [152] reported that methanolic extracts of *Jatropa curcas* L. were more effective than the aqueous and chloroform fractions in inhibiting phospholipase A_2_ activity. The authors attributed this to the possible presence of divalent ions (Calcium (II), Strontium (II), and Barium (II) ions) or quercetin-like compounds which are reported to augment the activity of phospholipase A_2_ through induction of conformational changes in its substrate-binding sites [167, 168]. Table 3 summarizes some of the solvents employed by studies done on antivenom activity of some plants reported in this survey. It is worth noting that methanol appears to be the solvent of choice probably because of its ability to dissolve both polar and non-polar compounds [169, 170].

Testing for the efficacy of plants as antivenins has been perfected using mice as the test specimens. Experimentally, the extracts are tested against the lethal dose of the venom that causes death of 50% of the subjects (LD_50_). Tests are done either in vivo or in vitro on specific toxic activities of venoms. So far, the inhibitory activity of most extracts has been tested against phospholipase A_2_, one of the toxic constituents of snake venoms [111].

## Conclusions and recommendations

Uganda has over 125 districts hence less than 1% of the country have been surveyed for antivenin plants. The inventory of plants utilized by Ugandan communities present considerable potential for the treatment of snake envenomation. The present review therefore opens the lead for isolation and elucidation of the chemical structures of the antivenom compounds from the claimed plants that could be harnessed in combined therapy with commercial antiserum. There is a need for concerted efforts by scholars, traditional healers, local authorities, and the state to address the ongoing African snakebite crisis and meet World Health Organizations’ great interest in documenting the various medicinal plants utilized by different tribes worldwide.

## Supplementary information

**Additional file 1.** Family, local name, botanical name, growth habit, conservation status, part used, method of preparation and route of administration of antivenin plants used in different districts of Uganda.

## Data Availability

This is a review article and no raw experimental data was collected. All data generated or analyzed during this study are included in this published article.

## Abbreviations

DPPH: 1,1- diphenyl 2-picrylhydroxyl
DPPH-1,1: Diphenyl 2-picrylhydroxyl
LD_50_: Median lethal dose
*N. naja*: *Naja naja*
PLA_2_: Phospholipase A
spp: Species
*V. russelli*: *Viper russelli*
